# Supplementary material for: A comprehensive survey of genetic variation in 20,691 subjects from four large cohorts
Source: PLoS One. 2017 Mar 16;12(3):e0173997. doi: 10.1371/journal.pone.0173997 (PMC5354293; doi:10.1371/journal.pone.0173997)
Supplement: S2 Fig — Different colors correspond to different imputation quality score r-sq thresholds. Data is categorized by minor allele frequency. (PDF) [file pone.0173997.s002.pdf]

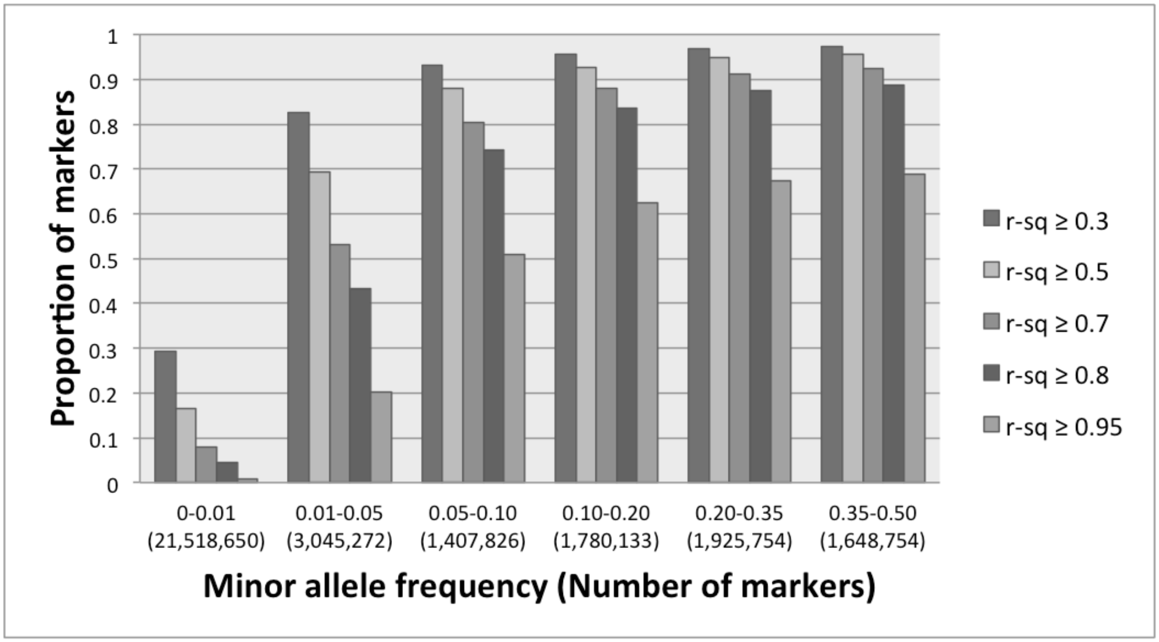

S2 Fig. Proportion of sucessfully imputed markers on the Illumina HumanHap platform. Different colors correspond to different imputation quality score r-sq thresholds. Data is categorized by minor allele frequency.
