# Supplementary material for: A comprehensive survey of genetic variation in 20,691 subjects from four large cohorts
Source: PLoS One. 2017 Mar 16;12(3):e0173997. doi: 10.1371/journal.pone.0173997 (PMC5354293; doi:10.1371/journal.pone.0173997)
Supplement: S4 Fig — A: QQ-plot for GWAS analysis of body mass index on the Illumina Omniexpress platform (n = 5,844). B: QQ-plot for GWAS analysis of body mass index on the Affymetrix platform (n = 7,677). C: QQ-plot for GWAS analysis of body mass index on the Illumina HumanHap platform (n = 6,762). (PDF) [file pone.0173997.s004.pdf]

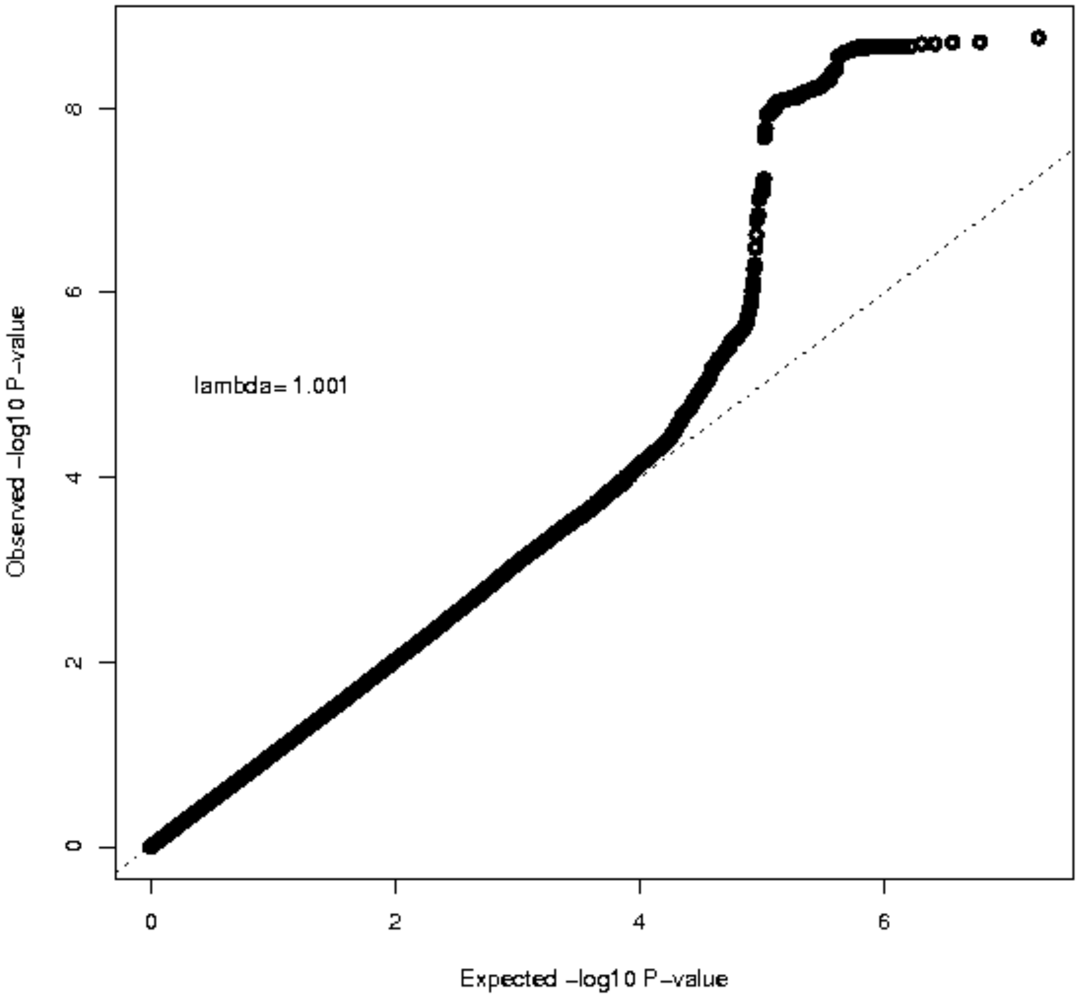

S4a Fig. QQ-plot for GWAS analysis of body mass index on the Illumina Omniexpress platform (n=5,844).

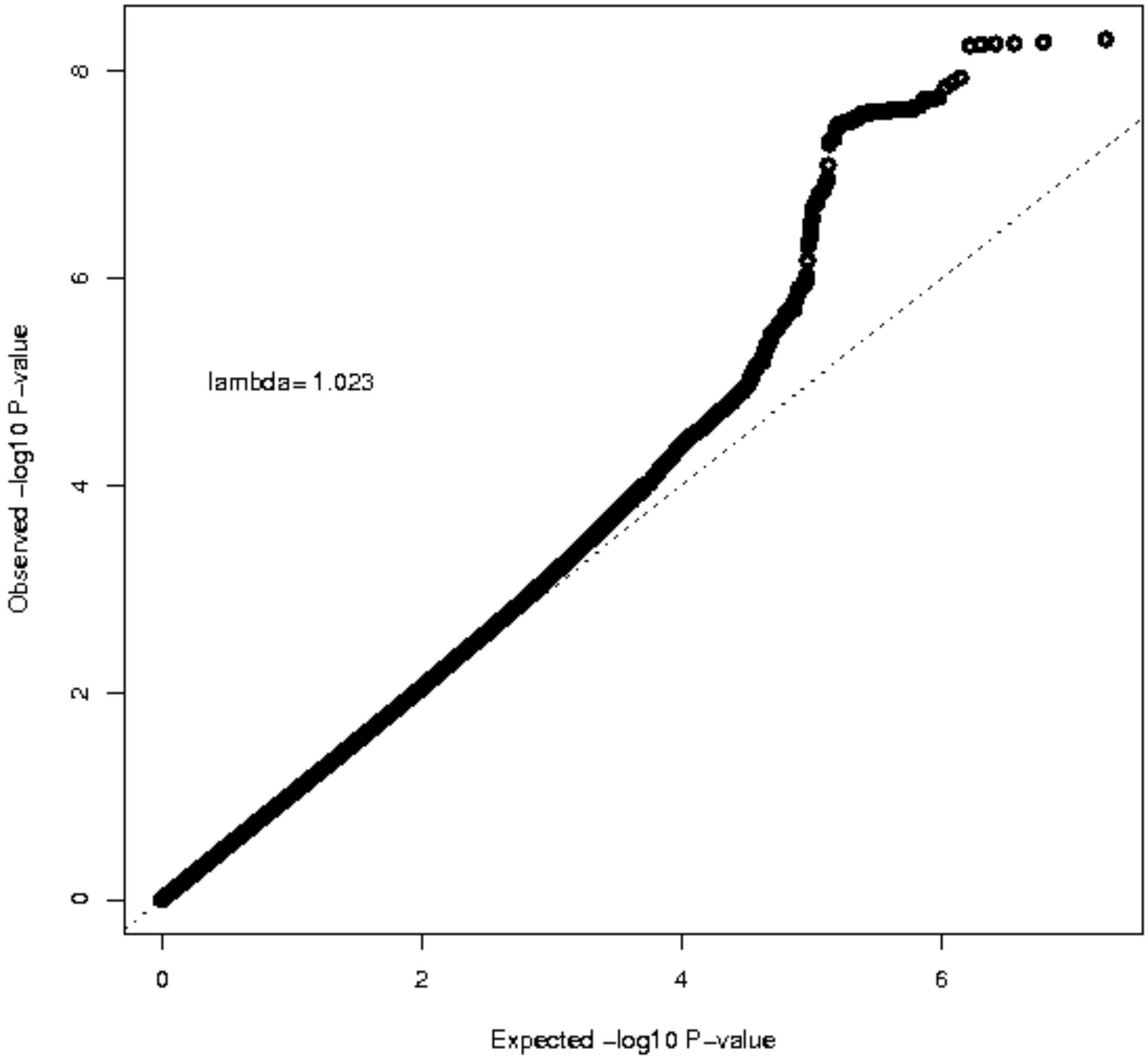

S4b Fig. QQ-plot for GWAS analysis of body mass index on the Affymetrix platform (n=7,677).

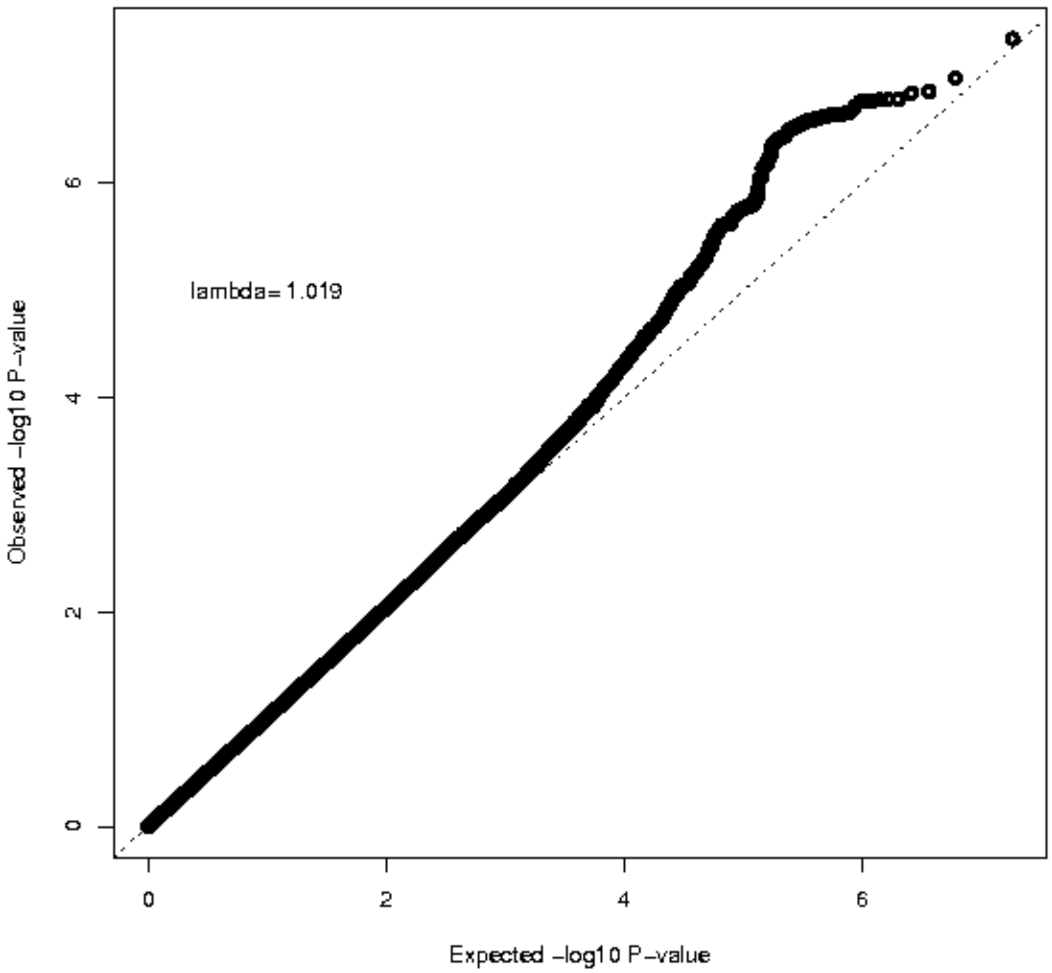

S4c Fig. QQ-plot for GWAS analysis of body mass index on the Illumina HumanHap platform (n=6,762).
