# Supplementary figures and images for: A comprehensive survey of genetic variation in 20,691 subjects from four large cohorts
Source: PLoS One. 2017 Mar 16;12(3):e0173997. doi: 10.1371/journal.pone.0173997 (PMC5354293; doi:10.1371/journal.pone.0173997)

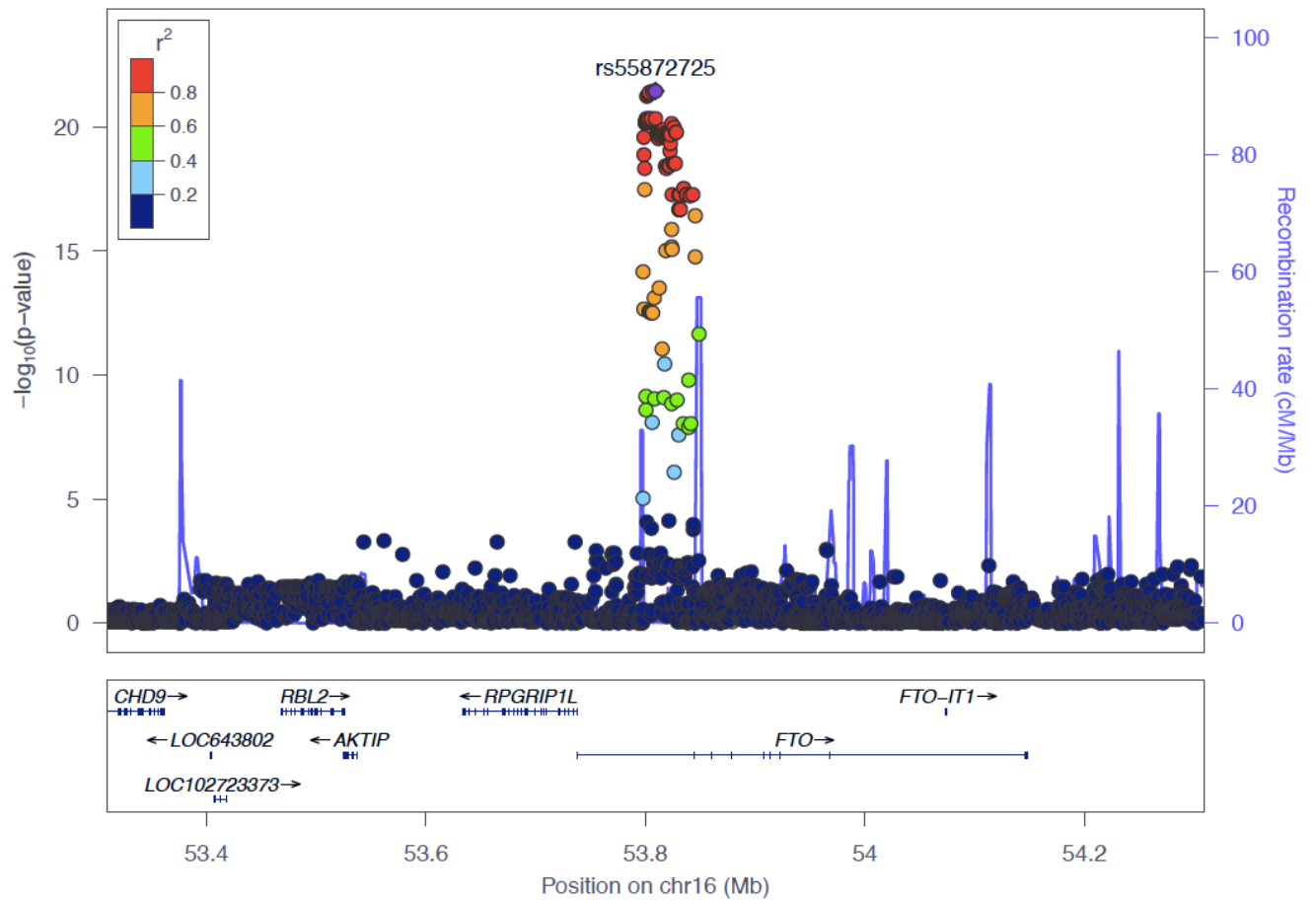

S5a Fig. LocusZoom plot for the BMI *FTO* locus.



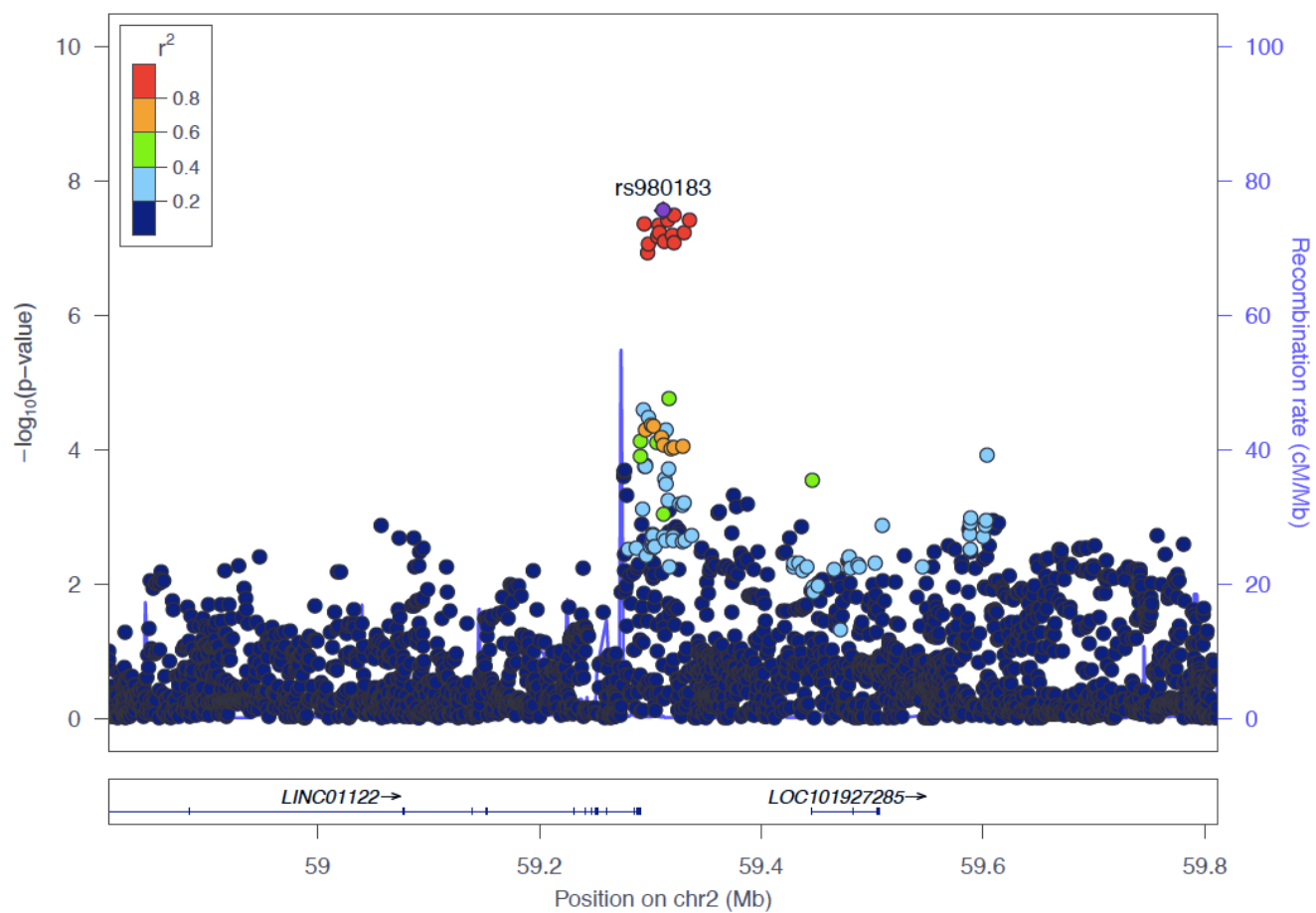

S5c Fig. LocusZoom plot for the BMI *FANCL* locus.

Supplement: S5 Fig — A: LocusZoom plot for the BMI FTO locus. B: LocusZoom plot for the BMI TMEM18 locus. C: LocusZoom plot for the BMI FANCL locus. (PDF) [file pone.0173997.s005.pdf]

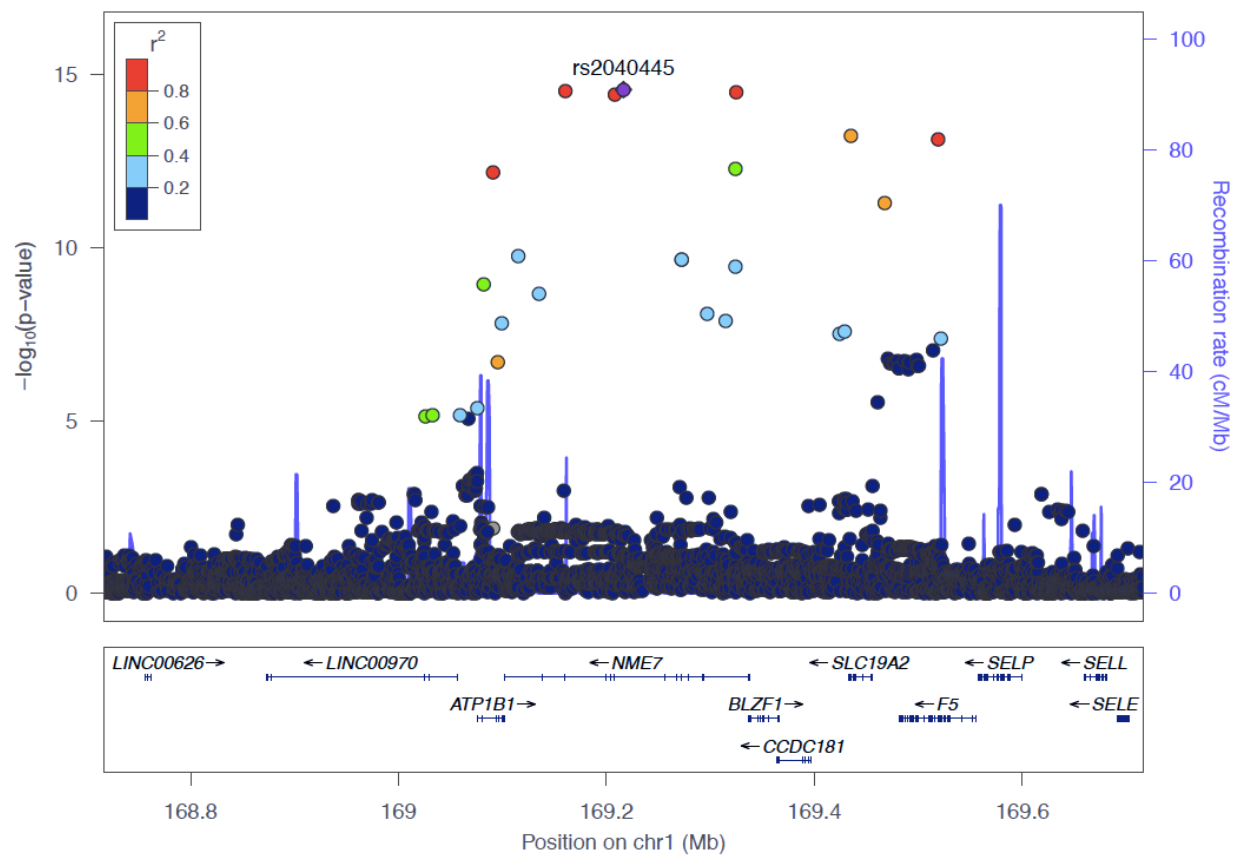

**S7a Fig. LocusZoom plot for the VTE F5 locus.**

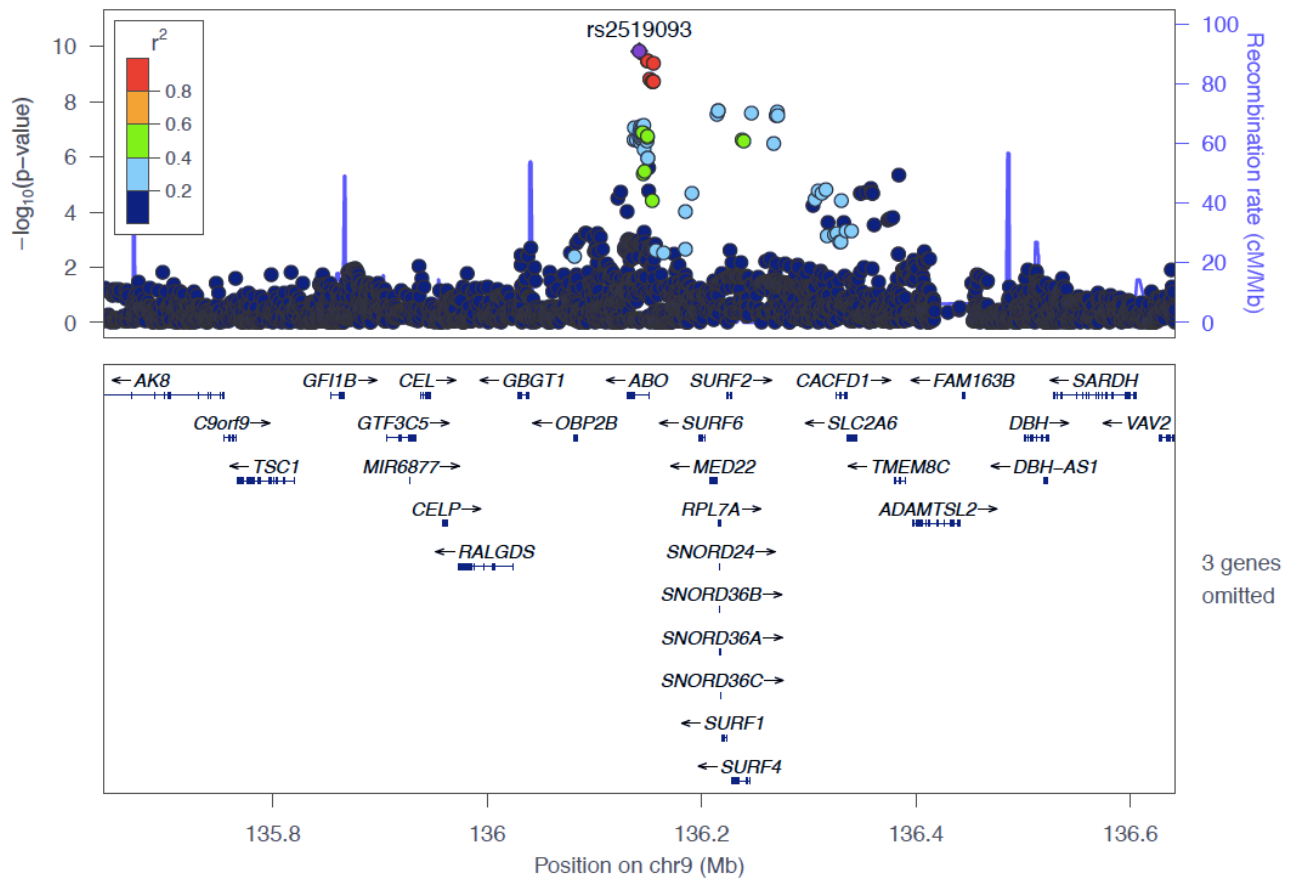

**S7b Fig. LocusZoom plot for the VTE *ABO* locus.**

Supplement: S7 Fig — A: LocusZoom plot for the VTE F5 locus. B: LocusZoom plot for the VTE ABO locus. (PDF) [file pone.0173997.s007.pdf]
