# Supplementary material for: A comprehensive survey of genetic variation in 20,691 subjects from four large cohorts
Source: PLoS One. 2017 Mar 16;12(3):e0173997. doi: 10.1371/journal.pone.0173997 (PMC5354293; doi:10.1371/journal.pone.0173997)
Supplement: S6 Fig — A: QQ-plot for GWAS analysis of venous on the Illumina Omniexpress platform (406 cases and 4,786 controls). B: QQ-plot for GWAS analysis of venous on the Illumina Omniexpress platform (406 cases and 4,786 controls). C: QQ-plot for GWAS analysis of venous on the Affymetrix platform (532 cases and 7,147 controls). (PDF) [file pone.0173997.s006.pdf]

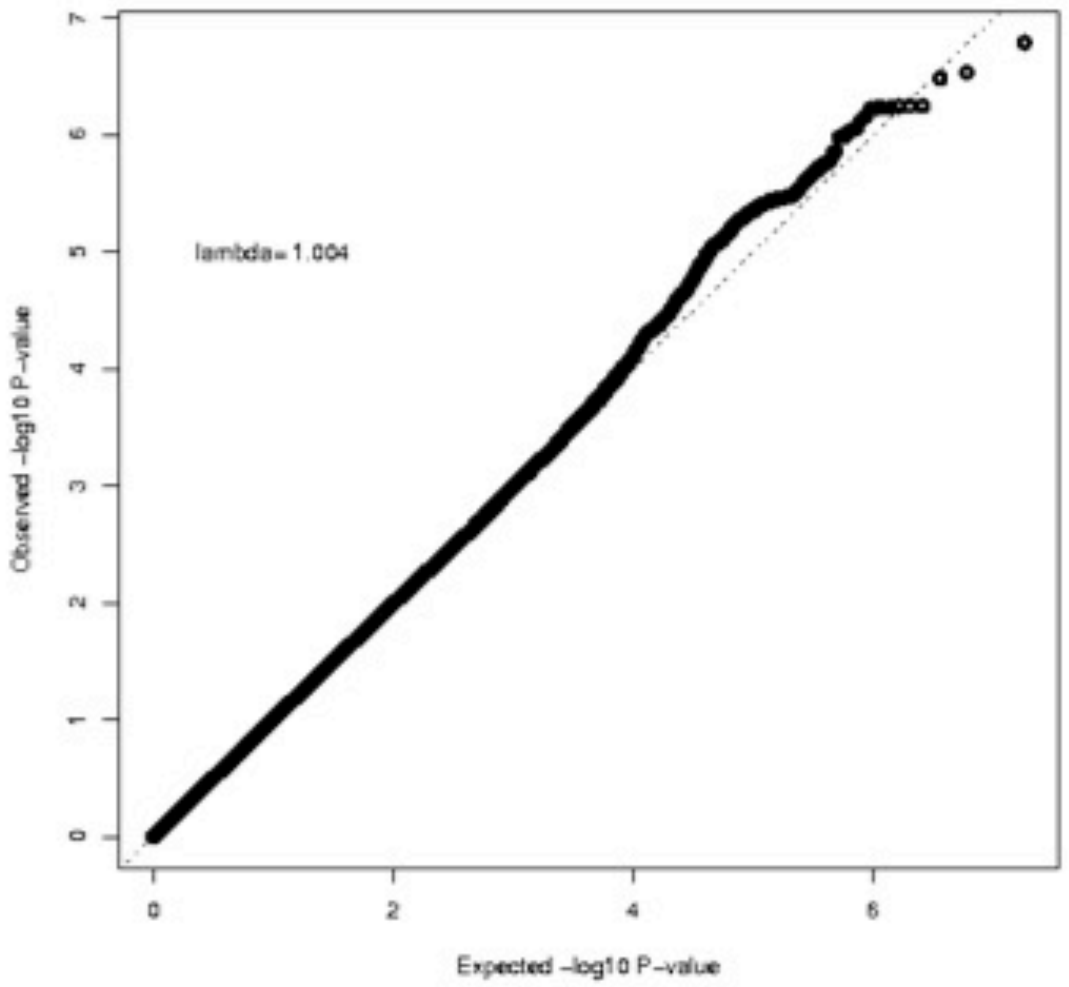

S6a Fig. QQ-plot for GWAS analysis of venous on the Illumina Omniexpress platform (406 cases and 4,786 controls).

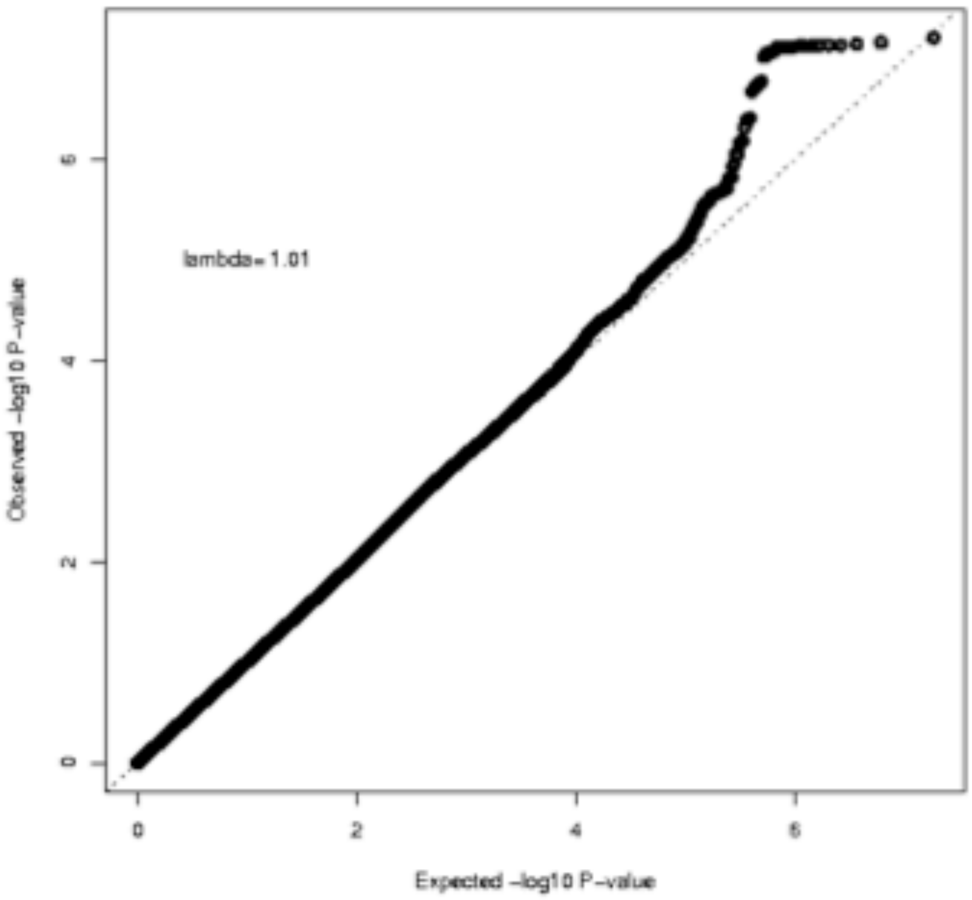

S6b Fig. QQ-plot for GWAS analysis of venous on the Illumina Omniexpress platform (406 cases and 4,786 controls).

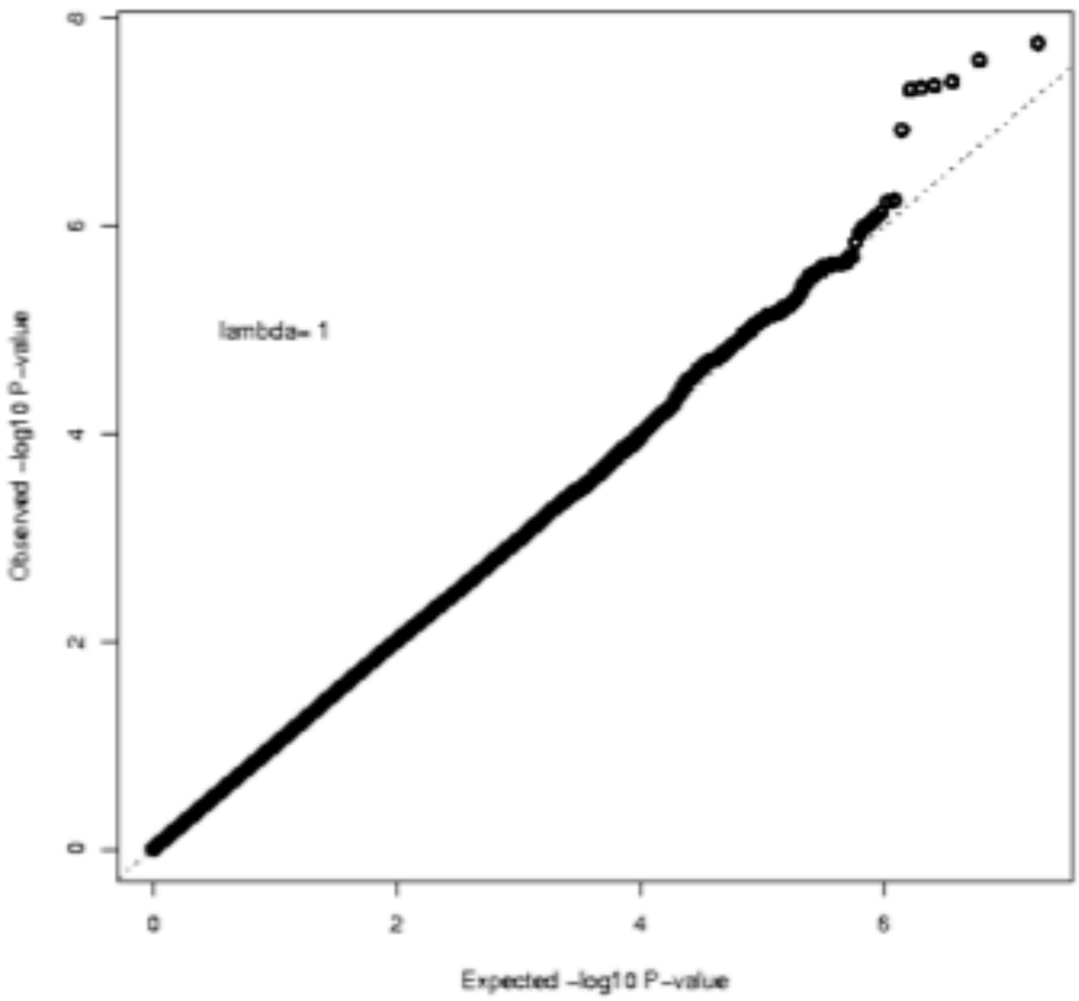

S6c Fig. QQ-plot for GWAS analysis of venous on the Affymetrix platform (532 cases and 7,147 controls).
