# Supplementary material for: A comprehensive survey of genetic variation in 20,691 subjects from four large cohorts
Source: PLoS One. 2017 Mar 16;12(3):e0173997. doi: 10.1371/journal.pone.0173997 (PMC5354293; doi:10.1371/journal.pone.0173997)
Supplement: S1 Table — (PDF) [file pone.0173997.s008.pdf]

S1 Table. Number of SNPs (N) with MAF ≤ 0.01 overall and by imputation quality score (r-sq) threshold for the three platforms Illumina HumanHap, AffyMetrix 6.0 and Illumina Omniexpress.

Platform	N	mean(r-sq)	N (%) r-sq≥0.3	r-sq≥0.5	r-sq≥0.8	r-sq≥0.95
Illumina HumanHap	21,518,650	0.224	6,279,290(29.2%)	3,558,835(16.5%)	983,575(4.6%)	162,938 (0.8%)
Affymetrix 6.0	21,542,876	0.241	6,721,060(31.2%)	4,079,981(18.9%)	1,375,400(6.4%)	298,816 (1.4%)
OmniExpress	21,554,521	0.243	6,885,575(31.9%)	4,170,866(19.4%)	1,342,897(6.2%)	270,436 (1.3%)
