# Supplementary material for: A comprehensive survey of genetic variation in 20,691 subjects from four large cohorts
Source: PLoS One. 2017 Mar 16;12(3):e0173997. doi: 10.1371/journal.pone.0173997 (PMC5354293; doi:10.1371/journal.pone.0173997)
Supplement: S2 Table — (PDF) [file pone.0173997.s009.pdf]

S2 Table. Associations for previously reported lead SNPs in regions that were genome-wide significant in analysis based on 20,283 individuals (BMI) and 1,364 cases and 17,628 controls (VTE) in NHS, NHSII, HPFS and PHS.

Trait	SNP	Chr	Pos	Effect allele	Effect allele frequency	Effect	P
BMI	rs1558902	16	53803574	a	0.4204	0.4525	$4.30x10^{-22}$
BMI	rs13021737	2	632348	a	0.1832	-0.3204	$6.88x10^{-8}$
BMI	rs1016287	2	59305625	t	0.2953	0.201	$7.68x10^{-5}$
VTE	rs4524	1	169511755	t	0.7371	0.0568	0.22
VTE	rs6025	1	169519049	t	0.0284	0.7533	$7.41x10^{-14}$
VTE	rs529565	9	136149500	t	0.6576	-0.2154	$1.80x10^{-7}$
