# Supplementary material for: A comprehensive survey of genetic variation in 20,691 subjects from four large cohorts
Source: PLoS One. 2017 Mar 16;12(3):e0173997. doi: 10.1371/journal.pone.0173997 (PMC5354293; doi:10.1371/journal.pone.0173997)
Supplement: S3 Table — (PDF) [file pone.0173997.s010.pdf]

S3 Table. Association with known VTE SNPs (Germain et al, AJHG 2015) based on our analysis including 1,364 cases and 17,628 controls from NHS, NHSII and HPFS

Lead SNP	Chr.	Gene	Risk allele	Risk allele frequency	Odds Ratio, Germain et al	P, Germain et al	Odds Ratio, this study	P, this study
rs6025	1	F5	T	0.033	3.25 (2.91–3.64)	1.10×10^{-96}	2.12 (1.74-2.59)	$7.41x10^{-14}$
rs4524	1	F5	T	0.736	1.20 (1.14–1.26)	2.65×10^{-11}	1.06 (0.97-1.16)	0.22
rs2066865	4	FGG	A	0.244	1.24 (1.18–1.31)	1.03×10^{-16}	1.05 (0.96-1.15)	0.27
rs4253417	4	F11	C	0.405	1.27 (1.22–1.34)	1.21×10^{-23}	1.13 (1.04-1.22)	$3.24x10^{-3}$
rs529565	9	ABO	C	0.354	1.55 (1.48–1.63)	4.23×10^{-75}	1.24 (1.14-1.34)	$1.80x10^{-7}$
rs1799963	11	F2	A	0.01	2.29 (1.75–2.99)	1.73×10^{-9}	1.52 (0.87-2.66)	0.14
rs6087685	20	PROCR	C	0.302	1.15 (1.10–1.21)	1.65×10^{-8}	1.05 (0.96-1.14)	0.31
rs78707713	10	TSPAN15	T	0.878	1.28 (1.19–1.39)	5.74×10^{-11}	1.13 (1.00-1.27)	$5.93x10^{-2}$
rs2288904	19	SLC44A2	G	0.785	1.19 (1.12–1.26)	1.07×10^{-9}	1.05 (0.95-1.15)	0.36
